# Initial Characteristics of Alkali–Silica Reaction Products in Mortar Containing Low-Purity Calcined Clay

**DOI:** 10.3390/ma17102207

**Published:** 2024-05-08

**Authors:** Daria Jóźwiak-Niedźwiedzka, Roman Jaskulski, Kinga Dziedzic, Aneta Brachaczek, Dariusz M. Jarząbek

**Affiliations:** 1 Institute of Fundamental Technological Research, Polish Academy of Sciences, Pawińskiego 5b, 02-106 Warsaw, Poland; kdzie@ippt.pan.pl (K.D.); aantolik@ippt.pan.pl (A.B.); djarz@ippt.pan.pl (D.M.J.); 2 Department of Civil Engineering, Wrocław University of Environmental and Life Sciences, Grunwaldzki Sq. 24, 50-375 Wrocław, Poland; roman.jaskulski@upwr.edu.pl

**Keywords:** alkali–silica reaction, ASR products, calcined clay, mortar, expansion

## Abstract

An alkali–silica reaction (ASR) is a chemical process that leads to the formation of an expansive gel, potentially causing durability issues in concrete structures. This article investigates the properties and behaviour of ASR products in mortar with the addition of low-purity calcined clay as an additional material. This study includes an evaluation of the expansion and microstructural characteristics of the mortar, as well as an analysis of the formation and behaviour of ASR products with different contents of calcined clay. Expansion tests of the mortar beam specimens were conducted according to ASTM C1567, and a detailed microscopic analysis of the reaction products was performed. Additionally, their mechanical properties were determined using nanoindentation. This study reveals that with an increasing calcined clay content, the amount of the crystalline form of the ASR gel decreases, while the nanohardness increases. The Young’s modulus of the amorphous ASR products ranged from 5 to 12 GPa, while the nanohardness ranged from 0.41 to 0.67 GPa. The obtained results contribute to a better understanding of how the incorporation of low-purity calcined clay influences the ASR in mortar, providing valuable insights into developing sustainable and durable building materials for the construction industry.

## 1. Introduction

An alkali–silica reaction (ASR) is a chemical process that results in the formation of a gel of alkali and alkaline earth metal silicates (mainly Na, K, and Ca) in concrete over time. This gel has the ability to increase its volume significantly by absorbing water. This leads to the formation of cracks and splits in concrete, weakening the structure of the material and reducing its tightness, which in turn reduces its durability.

An ASR is a multi-stage process. First, siloxane groups leach from the surface of the SiO_2_ crystals or amorphous silica that are present in the aggregate [1]. This process is favoured by the high pH of concrete pore solutions, and results in the formation of silanol groups which further react with OH^−^ ions. Silicate sols are then formed, polymerising with potassium (K^+^), sodium (Na^+^), and calcium (Ca^2+^) ions to produce, inter alia, ASR gel [2,3,4,5]. 

Three factors are required for the development of an ASR: a highly alkaline environment (Na^+^ and K^+^ ion concentrations above 0.22 mol/L and OH^−^ ions above 0.26 mol/L [2,6]), the presence of reactive forms of silica, and a humid environment. Therefore, one way to limit the development of an ASR is to eliminate one of these factors. This is usually achieved by using an aggregate that does not contain reactive forms of silica, or by reducing the amount of alkali, mainly by using low-alkali cements. 

The disadvantage of both of these methods is that they eliminate certain types of raw materials that are used in the production of cement and concrete. Alternative solutions are, therefore, still being sought. In recent years, considerable attention has been paid to the use of lithium compounds, especially lithium nitrate. However, even this method is not without its drawbacks. As the demand for lithium is expected to increase significantly due to the development of electromobility, its availability will decrease and its cost will increase proportionally. 

Another method for reducing an ASR is the use of supplementary cementitious materials (SCMs) [7,8,9,10,11]. These are either added to the concrete mix without reducing the amount of cement, or partially replace it. The use of SCMs reduces the ASR in two ways. The pozzolanic reaction of the SCM with portlandite results in the densification of the concrete. This reduces the mobility of the alkali ions and, therefore, their availability for the ASR. As a result of the same reaction, another proportion of alkali ions is incorporated into the resulting compounds. This also results in the formation of an ASR gel, but it is much more dispersed in the matrix, so that the local stress concentrations that damage the material matrix do not occur. 

The SCMs used in concrete technology, including for ASR reduction, are mostly industrial waste or by-products, such as fly ash, ground glass, and ground-granulated blast furnace slag [8,12]. Their availability will decrease as we move away from the use of coal for energy and steel production, and as a result of the implementation of closed-loop solutions. Therefore, research is also being carried out into the use of materials of natural origin that occur in large and evenly distributed deposits, such as clay. 

To maximise the potential of clay used as an SCM, it is subjected to a calcination process [13]. Calcined clay is increasingly being used in concrete technology due to its benefits [14,15]. Studies have confirmed that it can increase compressive strength [16,17] and reduce permeability [18]. The addition of calcined clay can also reduce the susceptibility of concrete to chloride ingress [18,19] and sulphate attack [20,21]. Calcined clay is also a promising component for reducing an ASR and its effects [22,23,24,25].

The best-known and most-intensively studied SCM among the group of calcined clays is metakaolin [26,27]. Metakaolin is formed by the calcination of kaolin, a clay that contains mainly kaolinite in its composition [28]. This process is carried out at temperatures in the range of 600 °C to 800 °C, resulting in the removal of the water that is chemically bound to the minerals that make up the clay. As a result of the calcination process, the minerals present in the clay largely lose their crystalline structure and take on an amorphous form. This process, known as dehydroxylation, involves the release of hydrogen from the OH groups in the mineral, which combines with the other hydroxyl groups to form water, which is then removed from the material [13,29]. In order to obtain a material with sufficiently high pozzolanic activity, the calcination time must not be too long, as this leads to recrystallisation and the formation of mullite [29]. It has been found [30] that aluminium-rich SCMs, such as metakaolin, are more effective at inhibiting ASR-induced expansion than pure silica SCMs. Two proposed mechanisms for the role that the aluminium contained in SCMs plays in controlling ASRs are as follows: the incorporation of aluminium into Calcium Silicate Hydrate (C-S-H), which enhances the alkali fixation capacity of the C-S-H; or, the presence of aluminium-rich SCMs increases the aluminium concentration of the pore solution, thereby reducing the silica dissolution rate and limiting the ASR [5,30].

Although metakaolin is an SCM with high pozzolanic activity and proven effectiveness at mitigating ASRs, the raw material from which it is formed, kaolinitic clay, is also used in many other industries, mainly in the paper industry, in the production of sanitary ceramics, and as an ingredient in paints, plastics, and rubber [31,32,33]. This, together with the fact that deposits containing clays with high kaolin content are not very numerous, has led to the interest of researchers in more common clays with a lower proportion of kaolin in their composition. As shown by the preliminary studies reported on in paper [34], they can also show significant effectiveness at mitigating ASRs, despite containing impurities, such as quartz, calcite, feldspar, mica, anatase, and sulphides, that affect pozzolanic activity [35]. However, the research on the efficacy of calcined clays of different qualities for ASR mitigation is still limited.

The characterization of ASR products is crucial to understanding the reaction mechanism and its impact on the performance properties of materials [36]. Various aspects, such as their composition, morphology, and distribution within the mortar matrix, contribute to an understanding of the ASR mechanism and its effects on the material’s performance. Aquino et al. [12] analysed the effect of metakaolin and silica fume on the chemistry of alkali–silica reaction products. They found a relationship between the size of expansion and the Ca/Si ratio. The results of their studies suggest that the calcium content in gel products may be related to swelling [12]. 

In their work, Gholizadeh-Vayghan et al. [36,37,38] investigated the effect of K/Si, Na/Si, and Ca/Si ion ratios on the swelling tendency of ASR gels. Their results suggest that increasing Na/Si and K/Si ratios have a positive effect on the swelling of gels, while Ca/Si ratios have a suppressing effect, except in the range between 0.18 and 0.40, where an increase in this ratio increases the swelling of gels. However, the calcium content of ASR gels does not significantly affect their equilibrium relative humidity [37]. The work of Hou et al. [39,40] investigated the swelling mechanism of ASR gels. Studies were carried out using field gel and cane mite as a synthetic model. The results of these studies made it possible to question one of the hypotheses regarding the mechanism of swelling of ASR gels, according to which it is the result of their layered structure that favours the accumulation of water in the space between the layers [38]. It was also shown that synthetically derived cane mite can be used to study the properties of ASR gels. Bektas and Wang [41] investigated the effect of ground clay brick on the chemistry of an ASR gel, and showed that the presence of this additive altered the chemistry of the ASR gel, resulting in a less expansive product.

Studying the composition of ASR products allowed scientists to identify the presence of swelling gels and the contribution they make to crack formation and expansion. The analysis of the morphology and distribution of ASR products has allowed for a better understanding of their effect on the microstructure and overall durability of the mortar.

This study aims to investigate the characteristics of the ASR products formed in mortar containing low-purity calcined clay. By evaluating the physical, chemical, and microstructural properties of the ASR products, we seek to provide insights into the potential impact of low-purity calcined clay on the long-term performance and durability of concrete. This research will contribute to the understanding of calcined clay as a sustainable option for mitigating ASRs, allowing for informed decisions regarding its optimal utilisation in concrete construction.

## 2. Materials and Methods

### 2.1. Raw Materials

**Figure 1:**
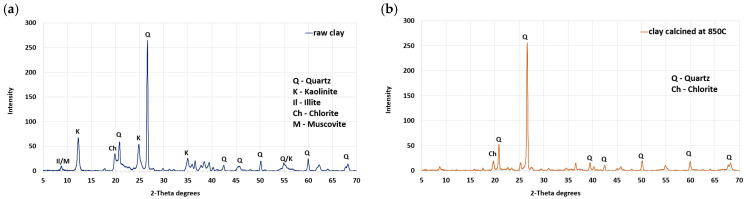
XRD patterns of (**a**) raw and (**b**) calcined clay.

To investigate the characteristics of ASR products modified with the addition of calcined clay, the ASTM C1567 [42] method was used to determine their impact on the expansion magnitude of the mortar bar specimens. A reference mortar without calcined clay, and mortars containing 10%, 20%, and 30% calcined clay as a substitute for cement were used. The research employed domestically common cement with the highest-available alkalis content, meeting standard requirements. Ordinary Portland cement CEM I 42.5R, with an alkali content of 1.12%, a Blaine’s specific surface of 546 m^2^/kg, and a MgO soundness of 0 mm determined via Le Chatelier test, was used. The raw clay (Quaternary clay from southern Poland, from the Beskids) employed had a 26% aluminium oxide (Al_2_O_3_) content, with a measured loss on ignition (LOI) of 9.20%, which classifies it as low-quality clay. Clay with a kaolinite content below 40% is commonly labelled as low-quality clay. The temperature range for the calcination of such clay typically spans from 700 °C to 900 °C [43]. In addition to kaolinite, muscovite, and illite, an X-ray diffraction (XRD) analysis identified quartz as one of the predominant minerals in the clay, as shown in Figure 1. 

Previous research [43] has shown that each clay mineral possesses a distinct optimal activation temperature. Specifically, kaolinite exhibits its optimal activation at 700 °C, palygorskite at 750 °C, montmorillonite at 800 °C, and illite at 850 °C. In the research we conducted, the clay was calcined at 850 °C. The clay underwent calcination in a laboratory chamber furnace boasting an 8.8 kW heating capacity. Before being placed in the furnace, the clay underwent drying at 110 °C until it reached a constant weight, followed by grinding and sieving through a 0.125 mm mesh. The heating rate was initially set at 4 °C/min until reaching approximately 150 °C, then it was increased to 12 °C/min thereafter. Once the temperature reached 850 °C it was sustained for 60 min, after which the material was permitted to cool in the oven until it reached a temperature below 50 °C. The cooling phase lasted at least 12 h. The detailed chemical and mineralogical composition of the raw clay and cement are presented in [22,34]. 

**Figure 2:**
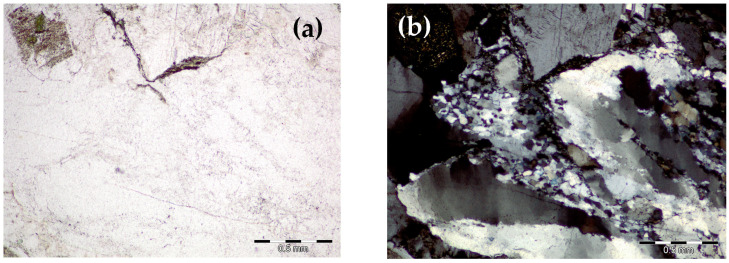
Granite petrography analysis: (**a**) parallel nicols, (**b**) crossed nicols.

All the mortars contained the same aggregate, which was previously examined and exhibited moderate reactivity (classified as aggregate reactivity class R1) [44,45]. The reactive aggregate used in this study was granite. Quartz and feldspar (including alkali feldspar and plagioclase) were found as the main phases. Also, biotite and amphibole were identified, as shown in Figure 2. The chemical composition obtained by the XRF method is presented in Table 1.

**Table 1:** X-ray fluorescence analysis (XRF) of granite aggregate (all values are in weight percent) [46].

Oxide Composition	SiO_2_	TiO_2_	Al_2_O_3_	Fe_2_O_3_	MnO	MgO	CaO	Na_2_O	K_2_O	P_2_O_5_	(SO_3_)
Weight percentage, %	72.6	0.22	15.01	1.47	0.04	0.34	1.38	4.16	3.78	0.07	<0.01

### 2.2. Specimens Preparation

Three sets of mortar bars, 25 mm × 25 mm × 285 mm, were prepared, each with a water-to-cement ratio (w/c) of 0.47. The mortar bars were cured for 24 h in steel moulds covered with a plastic coating to prevent drying, at a relative humidity greater than 90% and a temperature of 21 ± 1 °C. After removing the mortar bars from the steel moulds, they were described, and one end of each specimen was marked, indicating that it would be inserted into the measuring device from the top from that point forward. The specimens were dismantled and submerged in distilled water at 80 ± 2 °C for the next 24 h.

The specimens for the BSE mode were obtained by cutting the mortar bars with a low-speed diamond saw. The specimens, measuring 50 mm × 30 mm × 15 mm, were dried at 50 °C for 3 days, vacuum-impregnated with low-viscosity epoxy resin, lapped, and polished using a specialised procedure for SEM samples. Each specimen was prepared so that the polished surface tested was a cut from the centre of the mortar beam.

### 2.3. Test Methods

#### 2.3.1. Accelerated Mortar Bar Method

The research was conducted in accordance with the guidelines of the ASTM C1567 standard—Accelerated Mortar Bar Method [42]. The initial length measurements were recorded for each mortar bar after 24 h in 80 ± 2 °C distilled water, and the specimens were then exposed to 1 M NaOH at 80 ± 2 °C. The expansion of the mortar bars was systematically measured over a span of 14 days. An expansion of less than 0.10% after 14 days in the 1 M NaOH solution at 80 °C indicated satisfactory ASR mitigation. Nevertheless, the test was prolonged to 28 days for further analysis of the expansion curve.

#### 2.3.2. Scanning Electron Microscopy

The microstructure assessment was performed using a combination of a backscattered electron microscope (BSE) and an energy-dispersive X-ray (EDX) analysis of the specimens after ASTM C1567 testing. The analysis was conducted on specimens both with and without the addition of calcined clay. After polishing and lapping, the specimens were coated with carbon, and a strip of conductive tape was attached to each. Detailed tests were carried out using a JEOL JSM-6380 LA SEM-EDX device (Tokyo, Japan) at an accelerating voltage of 15 kV, an aperture 110 µm, and a working distance of 9–10 mm. The analysis of the chemical composition of the ASR gel was performed using an energy-dispersive X-ray spectroscopy, with a specific emphasis on the atomic ratios of Ca/Si, (Na+K)/Si, and Al/Si. The gel situated in the cracks of the aggregate and in the air voids was scrutinised. Roughly 80–100-point determinations of the chemical composition were carried out for each specimen.

#### 2.3.3. Nanoindentation

**Figure 3:**
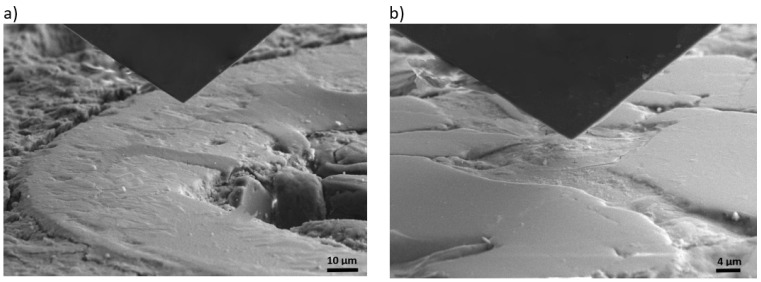
Exemplary locations of ASR gel indentations in (**a**) an air void and (**b**) a crack within the aggregate grains in the reference mortar without calcined clay.

The mechanical characteristics of the ASR products were assessed using the nanoindentation technique. Before each measurement, an SEM-EDX analysis was conducted to validate the composition and morphology of the ASR products. Nanohardness examinations were carried out with an in situ Alemnis indentation tester equipped with a diamond Berkovich tip, positioned within a Zeiss Crossbeam 350 enclosure (Oberkochen, Germany). A maximum force of 10 mN was applied, employing load control. The loading and unloading processes were executed at a rate of 0.2 mN/s. Following the attainment of maximum force, the indenter was held for 5 s. To ascertain the precise values of hardness and Young’s modulus from the indentation curves, the Olivier–Pharr method [47] was employed. The presented outcomes are the average of no less than 15 measurements for each mortar. The nanoindentations were performed at a distance that ensured the minimal influence of voids or other phases in the material. Furthermore, the indentation depth was in the range of 1 um, which ensured the study of the response of the minimum volume of material. The standard deviation of the results were lower than 5%. The distance between the imprints was five times greater than the size of the imprints. In Figure 3, microscope images are shown illustrating exemplary indentation sites in the ASR gel within the mortar in the air void, and in the crack within the aggregate grain. These illustrate that the indentation process occurred under precisely defined conditions in carefully selected locations to avoid undesired influences, such as being too close to the grain edge or the cementitious matrix. Before pressing the indenter, the chemical composition of the ASR gel was checked for identification purposes.

## 3. Results

### 3.1. Accelerated Mortar Bar Method

**Figure 4:**
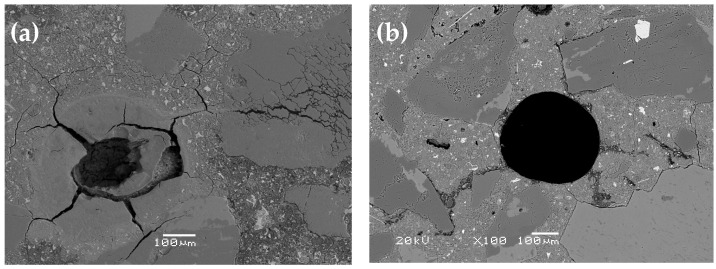
SEM-BSE micrograph of reactive aggregate particle and air void in mortars without additive (**a**) and with 30% calcined clay (**b**).

**Figure 5:**
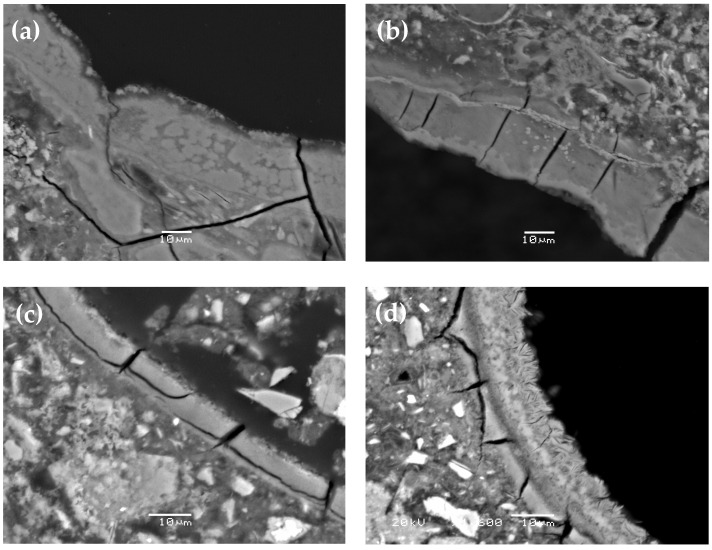
SEM-BSE micrograph of ASR products in air voids: (**a**) reference, (**b**) 10% calcined clay, (**c**) 20% calcined clay, and (**d**) 30% calcined clay.

The expansion results of the mortar bars, carried out in accordance with ASTM C1567 [42], are presented in Table 2. Clearly, after 14 days of testing, the mortar bars containing 20% and 30% calcined clay exhibited expansions below 0.1%. However, after 28 days, their results were comparable, approximately around 0.8%. At the same time, the reference mortar significantly exceeded the permissible limit of 0.1% expansion after 14 days, almost twice the value (0.196%), as well as the mortar with 10% calcined clay, which achieved an expansion of 0.165%. As a result of conducting the expansion study on mortar bars containing varying amounts of calcined clay, differences in the elongation of the specimens and the content of the products resulting from the ASR were observed, as shown in Figure 4 and Figure 5.

### 3.2. Microstructure Analysis

**Figure 6:**
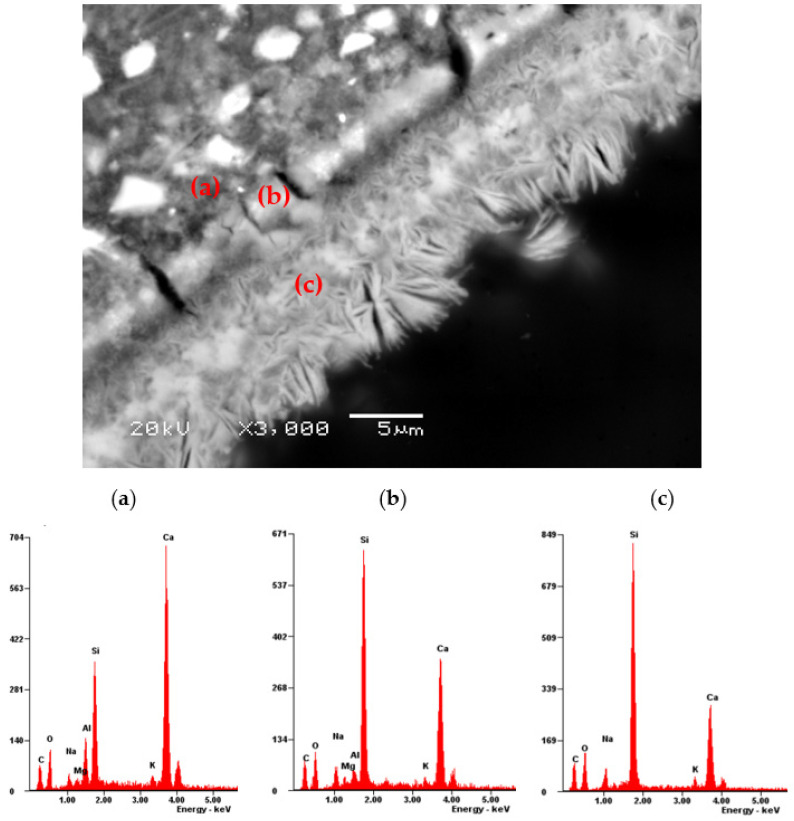
SEM-BSE micrograph with EDX analysis for the cement matrix and ASR products in an air voids in the mortar with 30% calcined clay: (**a**) cement matrix, (**b**) amorphous gel, and (**c**) crystalline gel.

During the analysis of the microstructure of the mortar specimens after testing according to ASTM C1567 [42], numerous cracks were observed in the aggregate grains and cement matrix. These cracks, like most air voids, were filled with alkali–silica reaction products, as shown in Figure 4. The most significant degradation of the mortar microstructure was observed in the reference specimens, and its severity decreased with the increasing content of calcined clay. A detailed microscopic analysis revealed differences in the morphology and chemical composition of the ASR products, as shown in Figure 5 and Figure 6, and Table 3 and Table 4. 

Crystalline and amorphous ASR products were found. As the content of calcined clay increased, the proportion of the crystalline form compared to the amorphous form of ASR gel also increased, as shown in Figure 5. Quantifiable measurements regarding the width of the ASR gel lining layer within the air voids are shown further in the article. There were also visible changes in the chemical composition of the ASR gel, as shown in Table 4. The amorphous gel was characterised by a higher content of Ca compared to the crystalline form of the gel, as shown in Figure 6. In the mortar bars containing calcined clay, the amorphous form of the ASR gel also contained small amounts of aluminium and magnesium.

The SEM-EDX analysis conducted on the ASR products, both in the aggregate grains and in the air voids within the cement matrix, revealed differences in the composition of the ASR gel resulting from the use of calcined clay, as shown in Table 3. Although the amount of added clay did not affect the composition of the ASR gel in the aggregate, there was a clear difference between the reference mortar (without clay) and the mortars containing clay. In the cement matrix, the ratio of Ca/Si and alkalis to Si decreased with an increase in the clay content. Also, differences in the composition of the ASR gel were evident concerning its form, whether amorphous or crystalline, as shown in Table 4. The amorphous form of the ASR gel was characterised by higher Ca/Si and Al/Si ratios compared to the crystalline form. However, a higher ratio of alkalis to Si was observed in the crystalline form of the ASR products. In the crystalline form of the ASR gel, there was virtually no aluminium, and there was significantly less magnesium.

### 3.3. Nanoindentation Test Results

After the SEM analysis and the precise identification of the locations for further investigation, mechanical properties testing of the ASR products was conducted using nanoindentation. Due to the absence of the crystalline form of the ASR gel in the reference mortar and its negligible quantity in the mortar containing 10% calcined clay, the indentation tests were conducted on the amorphous form of the ASR products. The indentation tests were also conducted on the aggregate grains (quartz) to relate the obtained results to the literature data, as shown in Table 5.

## 4. Discussion

The relationship between the expansion of the mortar bars and the content of low-quality calcined clay, as a substitute for mass cement, are shown in Table 2. The obtained results align with expectations, assuming that calcined clay displays pozzolanic properties as a supplementary cementitious material [34,48]. A comparable relationship was observed when utilising high-quality calcined clay, such as metakaolin [24,49].

The cause of the observed changes in the expansion of the specimens containing varying amounts of calcined clay was confirmed during our microscopic analysis, as shown in Figure 4, Figure 5 and Figure 6. The relationship between ASTM C1567 [42] expansion and the Si/Al ratio in the ASR gel concerning the content of calcined clay [22] as a cement substitute was confirmed. 

**Figure 7:**
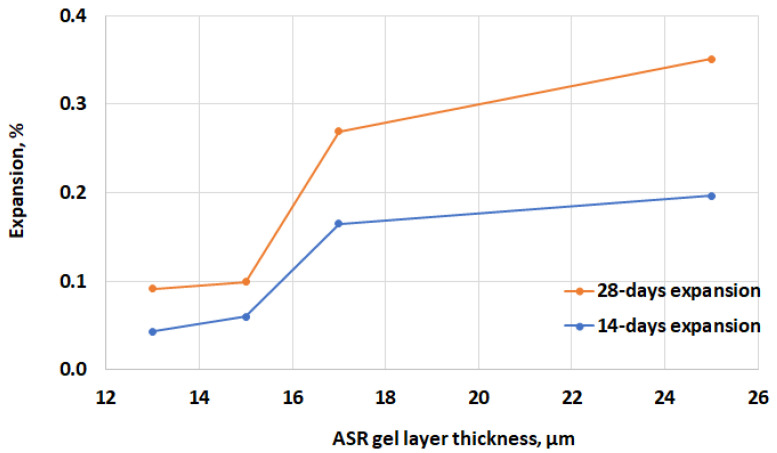
The relationship between the ASR gel thickness in the air voids and the expansion of the mortar bar specimens.

Based on our current and previous calculations [22] regarding the quantity of ASR gel (ASR gel thickness in air voids), it has been observed that 15 µm is a critical value, beyond which the expansion of the mortar bars significantly increases, as shown in Figure 7. 

As in the case of the investigation of the ASR gel properties in the aggregate grains [42], the movement from the edge of the air void towards its interior (from the cement matrix) resulted in a change in the structure of the ASR gel. It transformed from amorphous to crystalline with a decreasing calcium content. The decreasing of the ratio of Ca/Si and (Na+K)/Si with the increase in clay content may have also resulted from the dilution of the pore solution, due to the reduced amount of cement. The amorphous ASR gel creates narrow coatings, typically a few micrometres thick, along the crack surfaces, extending several hundred micrometres. In this area, they usually coexist with crystalline ASR products, which begin to fill the cracks or air voids as they propagate from the outer edges of the aggregate towards the centre [3].

Amorphous materials have a higher solubility compared to crystalline phases, allowing them to more effectively incorporate calcium ions from the surrounding environment. The solubility of a compound in the amorphous form is higher than the more stable crystalline form because the Gibbs free energy is higher [50].

The expansive properties of an ASR gel depend on its chemical composition [51]. The ASR gel identified in the cracks in the aggregate grains was characterised by a similar calcium content, regardless of the content of the calcined clay used. The Ca/Si ratio ranged from 0.20 to 0.24 for the mortars containing calcined clay. According to the literature [38], ASR gels with this composition have demonstrated enhanced swelling capacity and water absorption. Conversely, the ASR gel found in the air voids exhibited a higher calcium content (Ca/Si ratio ranging from 0.50 to 0.66), suggesting a diminished expansion potential [38,52]. While it was found that the ASR gel in the air voids did not undergo expansion, its presence remains detrimental due to its impact on frost resistance [53].

The impact of calcined clay on the composition of the ASR products within the cement matrix is readily apparent, as seen in Figure 6 and Figure 7, in contrast to Li’s results [23,24]. His findings indicated that the chemical composition and mineral phase of calcined clays play a key role in mitigating the ASR. However, no significant differences were observed in the gel composition between the mixture—the reference one or the one containing calcined clay. This is probably due to the fact that Li et al. [23] analysed specimens containing only 10% calcined clay. 

The research we conducted shows that the calcined clay had a significant impact on the composition of the ASR gel in the cement matrix, but it had a relatively smaller impact on the composition of the ASR gel present in the cracked aggregate grains. This discrepancy may be attributed to the greater availability of aluminium in the clay calcined in the cement matrix. According to the results from the literature [23], it is known that among the various types of calcined clays, those with the lowest aluminium oxide content are the least effective at controlling ASR-induced expansion. 

**Figure 8:**
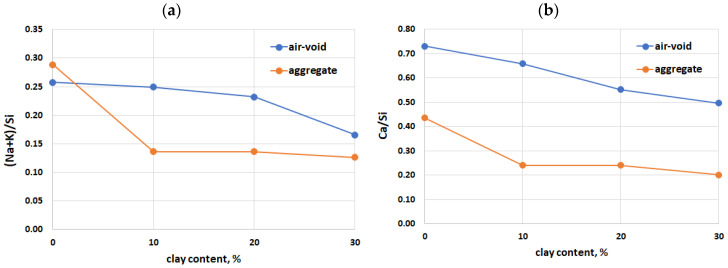
(Na+K)/Si ratio (**a**) and Ca/Si ratio (**b**) in ASR products as function of calcined clay content.

**Figure 9:**
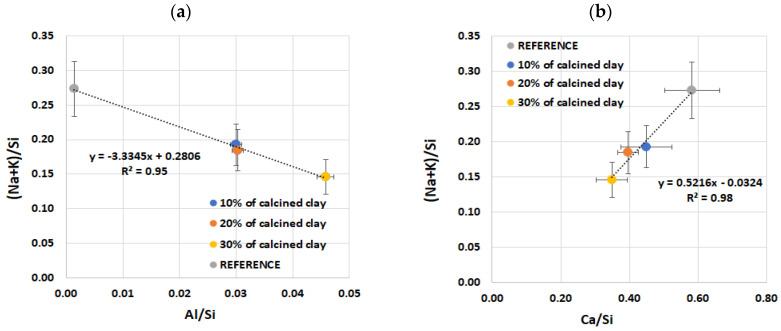
(Na+K)/Si ratio as a function of (**a**) Al/Si and (**b**) Ca/Si ratio of ASR products in air voids of investigated mortars without additive and with 10, 20 and 30% of calcined clay.

The content of (Na+K)/Si and Ca/Si in a cement matrix is clearly dependent on the content of calcined clay, as shown in Figure 8. As the content of calcined clay increases, concurrently with a reduction in the cement content, decreasing values of both dependencies are observed in the ASR gel within the cement matrix. It was also observed that the (Na+K)/Si ratio decreased as the Al/Si ratio increased, while it increased with the rise in Ca/Si, as illustrated in Figure 9. 

As mentioned earlier, in the crystalline form of the ASR gel there was no aluminium, and significantly less magnesium was present. The fact that an amorphous layer of ASR gel containing Al and Mg from the calcined clay formed first, followed by the appearance of a crystalline layer of ASR gel, may indicate the sequential evolution process of the ASR gel structure during an alkali–silica reaction. This phenomenon could suggest that, initially, a reaction occurred leading to the formation of an amorphous layer containing aluminium and magnesium from the calcined clay. Subsequently, in later stages of the process or under subsequent conditions, this amorphous layer could undergo crystalline transformations, ultimately forming the crystalline layer of the ASR gel. While Leemann et al. [54] discovered that the presence of aluminium in the pore solution of cement-based composites only has the capability to decelerate SiO_2_ dissolution without altering the morphology, structure, or composition of the reaction products, Krüger et al. [55] revealed that the addition of aluminium to the synthesis of alkali–silica gels induces a modification in the alkali–silica gel structure, due to the incorporation of aluminium into the silicate network. 

Souza and Sanchez [56] analysed the influence of various SCMs to avoid or mitigate ASR-induced expansion, and they examined the Vickers microhardness profile of the ASR products within the aggregate grains, and of the ASR gel within the aggregates/ASR gel at the edge of the aggregate/ASR gel in the binder paste using 98 mN. They showed that the ASR gel demonstrated lower Vickers hardness values when it was within the aggregate particles compared to when the gel was in contact with the cement matrix. However, at the interface between the aggregate/ITZ, the highest HV values were observed. They concluded that the higher the replacement level, the lower the Vickers hardness of the ASR gel found near the binder paste. However, the research we conducted showed opposite results: the higher the level of cement replacement, the higher the nanohardness values.

**Figure 10:**
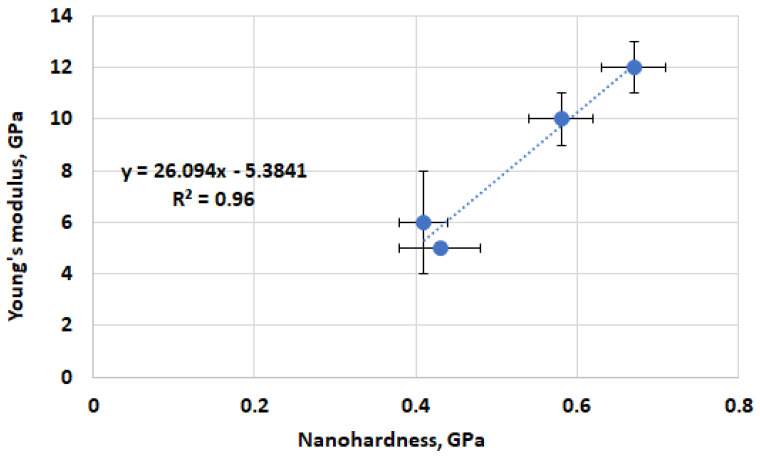
Young’s modulus vs. nanohardness of ASR products (amorphous) of investigated mortars without additive and with 10%, 20%, and 30% calcined clay.

The relationship between Young’s modulus and the nanohardness of the ASR products (amorphous) of the investigated mortars (Figure 10) fits into the trend seen in the literature [57]. Despite the fact that Leemann’s and Lura’s studies [57], as well as Wu et al.’s [58], were carried out on ASR gels found in aggregates, the present research focuses on ASR gel situated within air voids. The reaction product of the ASR within the aggregates of 45-year-old concrete from a bridge [57], as well as within the glass aggregate in the mortar bar specimens [58], were tested. The average values of Young’s modulus were 8.9 ± 1.0 GPa [57] and 17.9 ± 4.7 GPa [58], and Vickers hardness was 14.0 ± 2.3 [57], while the nanohardness was 0.50 ± 0.24 GPa [58]. Nevertheless, despite the diverse locations of the ASR products, the obtained results (Figure 7)—Young’s modulus ranging from 5 to 12 GPa and nanohardness from 0.41 to 0.67 GPa—align with the correlation between Young’s modulus and nanohardness which is known from the literature [57,58].

## 5. Conclusions

This study examined the effect of calcined clay on the microstructure and mechanical properties of alkali–silica reaction products, as well as on their chemical composition. Expansion studies, scanning electron microscopy with energy-dispersive X-ray spectroscopy, and nanoindentation tests were performed. The following conclusions can be drawn from the results obtained:

- The influence of calcined clay on limiting the expansion of the mortar bars as a result of the alkali–silica reaction of the aggregate was demonstrated.
- The microscopic analysis and chemical evaluations showed clear differences in the ASR products depending on the amount of calcined clay used. As the calcined clay content increased, the crystalline form of the ASR gel decreased.
- The ASR gel located in the air voids showed an average Ca/Si ratio of 0.73 ± 0.18 and (Na+K)/Si of 0.26 ± 0.04 for the reference specimens, and 0.50 ± 0.11 and 0.17 ± 0.02, respectively, for the specimens with 30% calcined clay content. The effect of the calcined clay dosage on the Al/Si ratio was minimal; however, adding calcined clay had an effect.
- The nanohardness increased with an increasing calcined clay content. Under a load of 10 mN, Young’s modulus of the amorphous ASR products ranged from 5 to 12 GPa, with a standard deviation of about 1 GPa, while the nanohardness ranged from 0.41 to 0.67 GPa, with a standard deviation of about 0.04 GPa.
- The determined values of Young’s modulus and nanohardness can be used for comprehensive numerical modelling of the mechanical properties of alkali–silica reaction products in concrete.

## Figures and Tables

**Table 2:** Expansion of the investigated mortar mixtures according to ASTM C1567 (%).

Mortar	Number of Days							
	4	7	11	14	18	21	25	28
Reference	0.041	0.085	0.145	0.196	0.254	0.297	0.334	0.351
10% calcined clay	0.033	0.081	0.138	0.165	0.207	0.243	0.263	0.269
20% calcined clay	0.009	0.022	0.056	0.060	0.081	0.088	0.096	0.099
30% calcined clay	0.007	0.020	0.034	0.043	0.062	0.065	0.078	0.080

**Table 3:** Composition of the ASR products depending on the place of occurrence.

Mortar	Ca/Si		Al/Si		(Na+K)/Si	
	Air Void	Aggregate	Air Void	Aggregate	Air Void	Aggregate
Reference	0.73 ± 0.18	0.43 ± 0.11	0.00 ± 0.00	0.01 ± 0.00	0.26 ± 0.04	0.29 ± 0.09
10% calcined clay	0.66 ± 0.15	0.24 ± 0.06	0.06 ± 0.00	0.00 ± 0.00	0.25 ± 0.02	0.14 ± 0.07
20% calcined clay	0.55 ± 0.12	0.24 ± 0.04	0.06 ± 0.00	0.00 ± 0.00	0.23 ± 0.02	0.14 ± 0.05
30% calcined clay	0.50 ± 0.11	0.20 ± 0.02	0.08 ± 0.00	0.01 ± 0.00	0.17 ± 0.02	0.13 ± 0.05

**Table 4:** Composition of ASR products in air voids.

Mortar	Ca/Si		Al/Si		(Na+K)/Si	
	Amorphous	Crystalline	Amorphous	Crystalline	Amorphous	Crystalline
Reference	0.73 ± 0.18	—	0.00 ± 0.00	—	0.26 ± 0.04	—
10% calcined clay	0.80 ± 0.04	0.52 ± 0.17	0.08 ± 0.02	0.04 ± 0.01	0.24 ± 0.01	0.25 ± 0.02
20% calcined clay	0.64 ± 0.13	0.46 ± 0.04	0.08 ± 0.02	0.04 ± 0.03	0.22 ± 0.04	0.24 ± 0.03
30% calcined clay	0.59 ± 0.29	0.40 ± 0.01	0.12 ± 0.07	0.04 ± 0.00	0.14 ± 0.02	0.19 ± 0.01

**Table 5:** The nanoindentation test results for the ASR products (amorphous) in air voids and in the aggregate grains at a constant force of 10 mN.

Mortar	Displacement, nm		Nanohardness, GPa		Young’s Modulus, GPa	
	Air Void	Aggregate	Air Void	Aggregate	Air Void	Aggregate
Reference	990 ± 30.0	304 ± 6.0	0.41 ± 0.03	2.0 ± 0.1	6 ± 2.0	88 ± 3.0
10% calcined clay	980 ± 50.0	-	0.43 ± 0.05	-	5 ± 0.2	-
20% calcined clay	840 ± 40.0	-	0.58 ± 0.04	-	10 ± 1.0	-
30% calcined clay	780 ± 20.0	-	0.67 ± 0.04	-	12 ± 1.0	-

## Data Availability

The raw data supporting the conclusions of this article will be made available by the authors on request.
